# Use of transanal irrigation (TAI) in the treatment of persistent bowel disorders in patients with endometriosis: A retrospective study

**DOI:** 10.1007/s00384-024-04757-x

**Published:** 2024-11-14

**Authors:** Simone Orlandi, Paolo Bocus, Andrea Geccherle, Giacomo Ruffo, Marcello Ceccaroni

**Affiliations:** 1 Department of Gastroenterology and Digestive Endoscopy, IRCCS Ospedale Sacro Cuore Don Calabria, Negrar di Valpolicella, Verona, Italy; 2 Department of Gastroenterology and Endoscopy, IRCCS Ospedale Sacro Cuore Don Calabria, Negrar di Valpolicella, Verona, Italy; 3 IBD Unit - IRCCS Ospedale Sacro Cuore Don Calabria, Negrar di Valpolicella, Verona, Italy; 4 General Surgery Unit, IRCCS Ospedale Sacro Cuore Don Calabria, Negrar di Valpolicella, Verona, Italy; 5 Department of Obstetrics and Gynecology, Gynecologic Oncology and Minimally-Invasive Pelvic Surgery, International School of Surgical Anatomy, IRCCS Ospedale Sacro Cuore, Negrar di Valpolicella, Verona, Italy

**Keywords:** Endometriosis, Transanal irrigation, Bowel dysfunction, Quality of life

## Abstract

**Purpose:**

Endometriosis has a strong impact on women’s quality of life (QoL). Pain is the main symptom of the disease, but bowel dysfunctions such as fecal incontinence, constipation and voiding difficulties are also reported. Patients could suffer from a Low Anterior Resection Syndrome (LARS)-like syndrome. Transanal irrigation (TAI), known to alleviate LARS-related symptoms, has been suggested to aid bowel dysfunction in endometriosis patients, potentially facilitating pelvic floor rehabilitation.

**Methods:**

We retrospectively collected data from 60 patients with endometriosis and bowel dysfunction who had been prescribed with the Peristeen^®^ Plus TAI system. Patient satisfaction, pain, QoL and LARS score were evaluated before (baseline) and after TAI treatment (follow-up of approximately 12 months).

**Results:**

Of the 60 patients meeting the inclusion criteria, 12 patients did not complete the questionnaires at follow-up and 16 patients discontinued treatment. Data analysis performed on 32 patients showed a mean increase of 3.6 points in patient satisfaction after TAI treatment and a mean pain reduction of 1.8 points (both, p-value < 0.001). LARS score decreased from a mean of 21.9 at baseline to a mean of 12.7 (p-value < 0.001). Accordingly, there was a notable improvement in patients’ QoL.

**Conclusion:**

TAI is a useful treatment for bowel dysfunctions in patients with endometriosis. When offered to these patients, TAI seems to represent a valuable strategy to reduce pelvic floor stress. This study confirms that TAI was associated to a significantly higher patients’ satisfaction, as well as to a reduction of pain and LARS-like symptoms.

## Introduction

Endometriosis is a chronic inflammatory disease that leads to the formation of endometriotic lesions [1]. According to the World Health Organization, it affects approximately 10% of women of reproductive age, who experience severely debilitating symptoms with a strong impact on their quality of life (QoL) [2, 3].

Several causes of endometriotic tissue have been postulated – e.g. retrograde menstruation, coelomic metaplasia, lymphatic and vascular metastasis [4–6] - but these theories alone do not explain all the epidemiological/pathophysiological features of the disease; other factors are involved in maintaining cell proliferation and the lesion, such as altered immunity and hormonal influences, as well as genetic, environmental and lifestyle factors [4, 5, 7]. However, it is clear that painful symptoms play a major role, as women with endometriosis often suffer from chronic pelvic pain, dysmenorrhea, deep dyspareunia, dysuria and dyschezia [8]. In addition, infertility occurs in approximately 30–50% of women with endometriosis [9], and concomitant irritable bowel syndrome (IBS) and/or constipation has been reported in 29% of cases [10]. Pelvic pain in women with deep infiltrating endometriosis (i.e. lesions > 5 mm deep) has recently been associated with hypertonia and difficulty in relaxing the pelvic floor muscles (PMF), which in turn may explain the presence of bowel symptoms [11].

Given the complexity of the condition, the treatment of endometriosis relies on various approaches, including pharmacological and surgical therapies [1, 4, 5]. In terms of pain management, the most widely accepted guidelines agree that progestogens and combined oral contraceptives should be used as first-line therapy [12]; in advanced stages of the disease, surgical removal of the endometriotic areas may be necessary. Whether conservative or radical surgery is performed depends on the extent to which the endometriotic implants are infiltrated and pain, with individualized treatment guided by implant location and type of pelvic pain, particularly in cases of deep infiltrating endometriosis, which often causes noncyclic chronic pelvic pain [13].

The positive effects of surgery on pain management can be offset by various postoperative complications, including low anterior resection syndrome (LARS)-like symptoms [14]. In fact, women with endometriosis may suffer from fecal incontinence and/or constipation, not only due to the disease itself and PMF dysfunction, but also as a result of pain management surgery [15]. Unfortunately, bowel symptoms associated with endometriosis are still poorly understood, and there are no guidelines for their treatment. In this regard, post-surgical pelvic floor physical therapy, although potentially beneficial, appears to be underappreciated and under-researched, limiting the availability of clinical evidence for its effectiveness in women with endometriosis [16].

Given the symptomatic analogies between patients with LARS and patients with endometriosis and bowel dysfunction, the beneficial effects of transanal irrigation (TAI) in the treatment of LARS and functional bowel dysfunction [17, 18] could also apply to the treatment of endometriosis. Indeed, TAI represents a minimally invasive and effective approach that allows patients to gain control over bowel function [19]. TAI allows the introduction of water into the bowel via the anus and, possibly thanks to consistent mechanical irrigation and/or stimulation of colonic movements, emptying of the rectosigmoid and left colon [20, 21]. Regular irrigation has been associated with a reduction in pain caused by bowel dysfunction and an improvement in QoL [17, 19]. As a strategy to reduce pelvic floor stress and facilitate rehabilitation, patients with endometriosis and bowel dysfunction referred to the Sacro Cuore Don Calabria Hospital underwent TAI treatment prior to pelvic floor physical therapy, to complement the overall management of bowel dysfunction in endometriosis. In this retrospective study, we aimed to evaluate the effectiveness of TAI in improving pain-related symptoms and QoL of patients with endometriosis combined with bowel dysfunction, with the primary goal of improving their clinical condition.

## Methods

The protocol of this retrospective observational study was approved by the Ethics Committee of the IRCCS Sacro Cuore Don Calabria (Negrar Di Valpolicella, Verona, Italy). The study was conducted in accordance with the principles of the Declaration of Helsinki (World Medical Association, 2013) and Good Clinical Practice for clinical investigations of medical devices in humans.

### Patients and visits

The study included 60 consecutive patients who met the following inclusion criteria: age over 18 years; diagnosis of endometriosis (at any stage of the disease); presence of specific bowel symptoms such as urge incontinence, constipation and voiding difficulties; prescription for Peristeen^®^ Plus Irrigation System (Coloplast, Humlebaek, Denmark), in the last five years (2018–2022), followed by appropriate training in the use of the device under the guidance of a specially trained therapist, and according to the manufacturer’s instructions. Patients who did not meet the above criteria were excluded from the analysis.

Patients were routinely subjected to a pretreatment visit (baseline) to record their medical history, prescribe the use of TAI with the Peristeen device and train them. Further visits were scheduled at approximately 4 weeks from baseline, to confirm the accuracy of the TAI procedure, and approximately 12 months after the start of the therapy (follow-up).

### Objectives and endpoints

The primary objective of the study was to analyze the subjective evaluation of pain and bowel symptoms 12 months after the start of the TAI treatment in patients with endometriosis – both operated and non-operated. The associated endpoints included a comparison of pain and satisfaction levels collected at baseline and follow-up, using for both evaluations a visual analog scale (VAS) ranging from 0 (absence of pain / not at all satisfied) to 10 (maximum pain intensity / completely satisfied).

Secondary objectives included: the assessment of LARS-like symptoms after TAI treatment by comparing the LARS scores - ranging from 0 (no LARS) to 42 (major LARS) – at baseline and follow-up [22] and an evaluation of the QoL after TAI treatment by comparing the Endometriosis Health Profile (EHP)-5 scores at baseline and follow-up. The questionnaire includes 5 items, which are rated on a five-points scale (never = 0, rarely = 1, sometimes = 2, often = 3, always = 4) [23].

Safety was assessed analyzing the frequency and severity of adverse events (AEs) using the Common Terminology Criteria for Adverse Events (CTCAE, version 5.0, 2017). Moreover, the reasons for discontinuing the treatment, as well as the use of laxatives or antidiarrheals, were recorded and analyzed.

### Statistical methods

Continuous variables were summarized as mean, median, standard deviation (SD), interquartile range (IQR), minimum and maximum; categorical variables were summarized as number (N) and percent of non-missing values. For non-normal data, the IQR was used instead of the SD. The normality assumption was tested using the Shapiro-Wilk test.

The null hypothesis that the values at follow-up did not change compared to baseline for the LARS score, the VAS for satisfaction and the VAS for pain scores was tested using the signed-rank test. The frequency distribution of responses to the EHP-5 questionnaire at baseline was cross-tabulated with the same response values at follow-up. Agreement between visits was estimated using the weighted kappa statistic and tested for significance using symmetry. All tests were two-sided and considered significant at the 5% level. All analyzes were performed using SAS 9.4. (NC, Cary)

## Results

This retrospective analysis included data from 60 consecutive female patients with a mean (SD) age of 44.3 (8.3) years (range, 33–68). 30% of women underwent segmental colon resection (including 5 cases with ileostomy and 1 case with colostomy, all of which had stoma closure performed years before TAI treatment), 13% had rectal shaving, and 5% had discoid resection. A smaller percentage had STARR surgery (5%), anterior rectal resection and ileostomy (2%), total colectomy (2%) and surgery for obstetric trauma (2%). Moreover, 46.9% of patients reported the use laxatives.

Concerning TAI (Table 1), most patients (56.0%) required an average of 30–45 min for the procedure, while only a minority (12.0%) required up to one hour; almost all patients (96.3%) didn’t require assistance. The mean (SD) volume of water used for irrigation was 571 (237) ml. Most patients (60.9%) performed TAI on alternate days (or three times a week), 17.4% once a day and 13% twice a week. Only 2 patients performed the procedure three times a day or more.

**Table 1:** TAI procedure details

TAI procedure		Statistics^*^
Duration in minutes	< 30	32.0%
	30–45	56.0%
	45–60	12.0%
Volume of water per procedure (in mL)		571 (237)
Frequency of the procedure	Daily	26.1%
	Alternate days (or 3/week)	60.9%
	Every 2 days (or 2/week)	13.0%

^*^ Statistics are mean (SD) for volume of water and number of irrigations, % otherwise

Moreover, 94% of patients reported that they were unable to void when not using the TAI system.

Of the included patients, 12 (20%) did not complete the questionnaire at follow-up and 16 (26.7%) discontinued the treatment. The reasons for discontinuation were the occurrence of symptoms/complications (*n* = 2; 3.3%), disappearance of symptoms or need for surgery (*n* = 3; 5.0%), ineffectiveness (*n* = 6; 10.0%), technical difficulties (*n* = 2; 3.3%) and personal reasons (*n* = 3; 5.0%). Accordingly, the data and questionnaires of 32 patients were analyzed at baseline and after a 12 months follow-up.

**Fig. 1:**
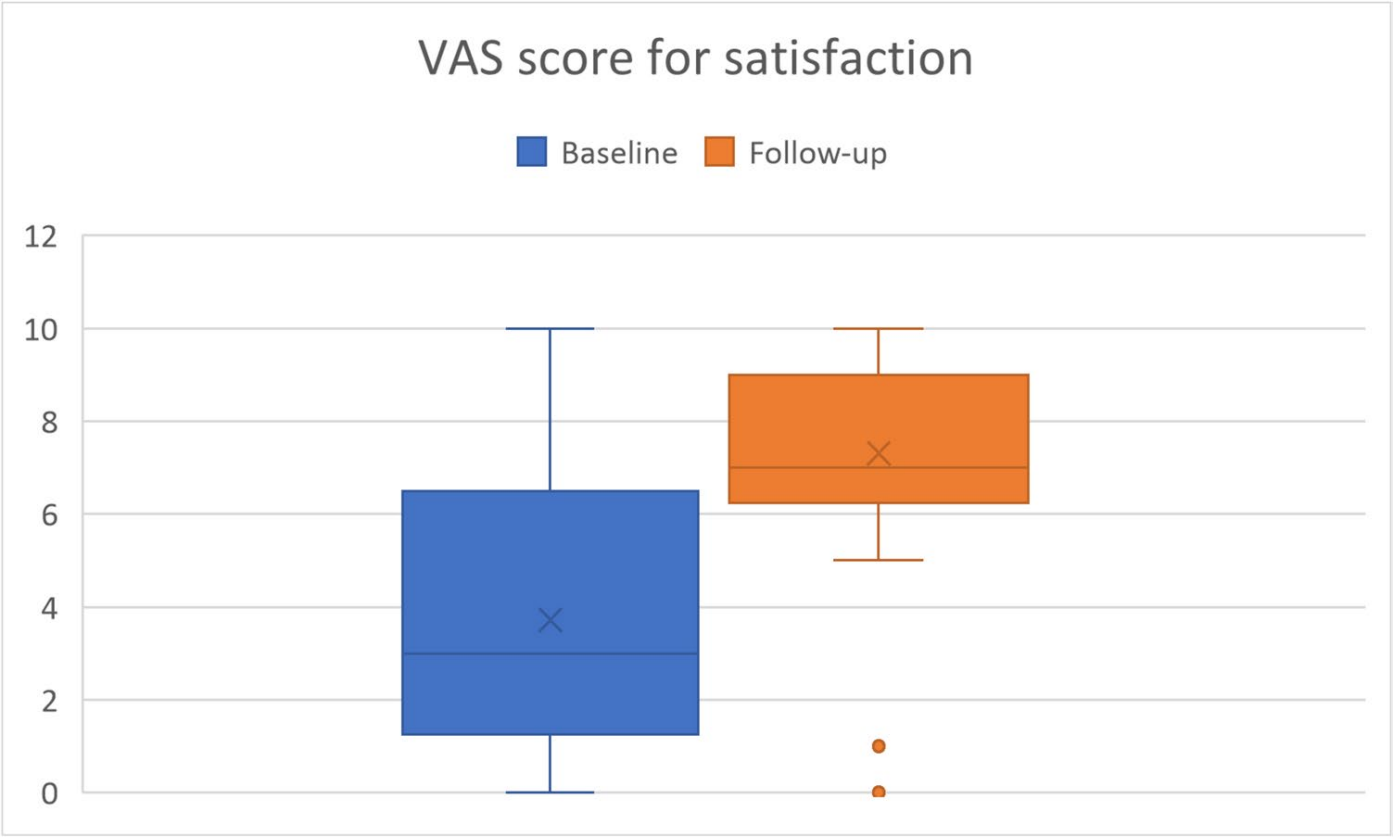
VAS for Satisfaction by visit

**Fig. 2:**
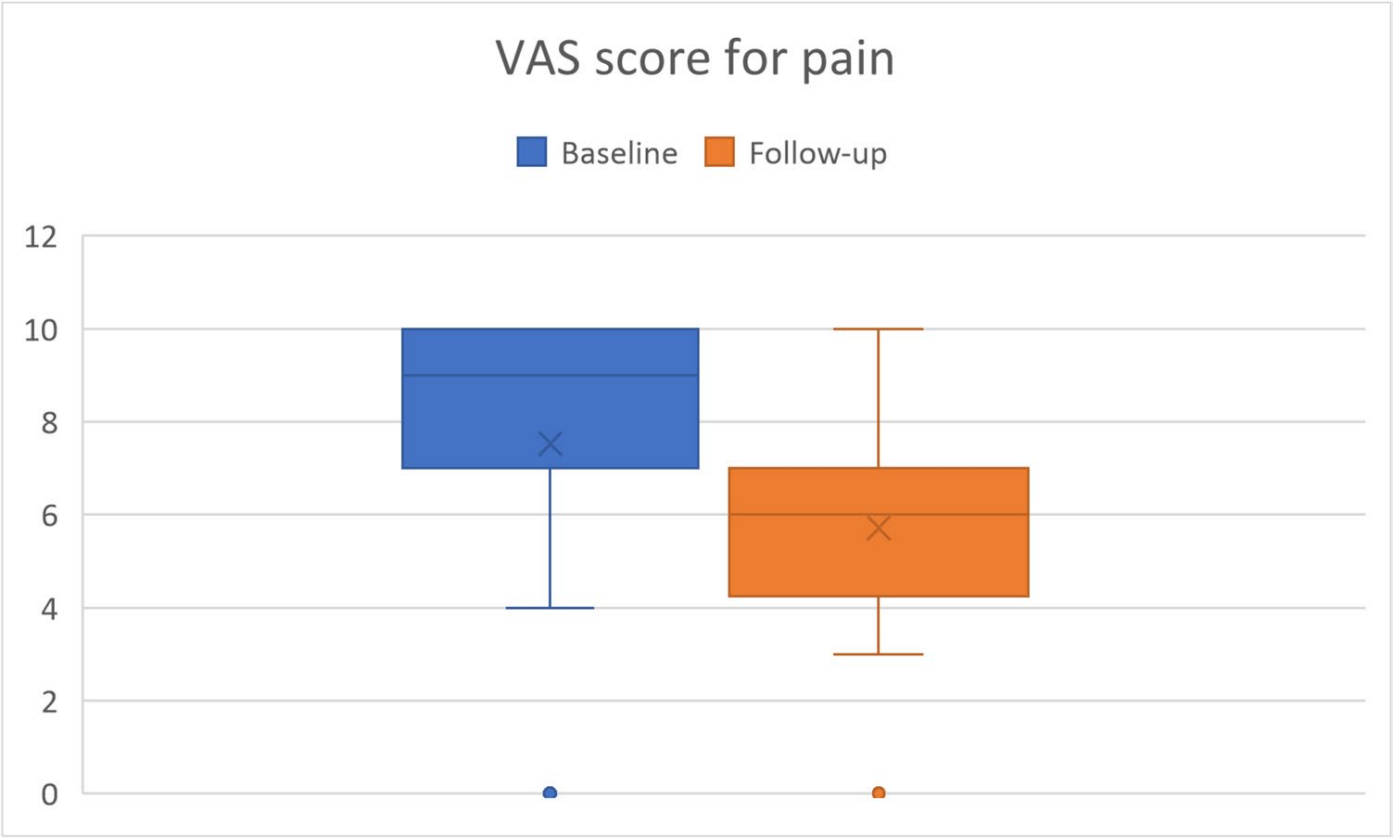
VAS for Pain by visit

**Fig. 3:**
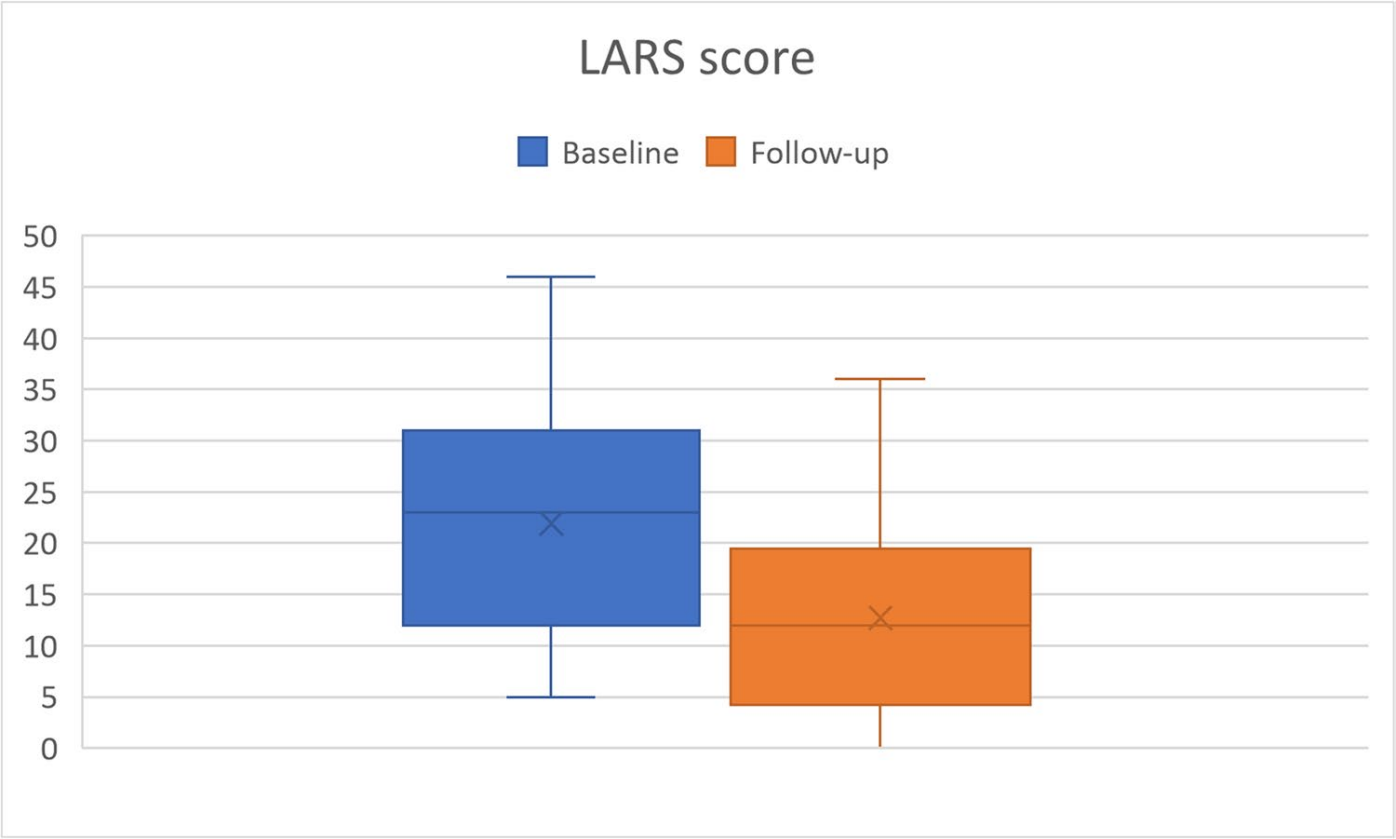
LARS score by visit

The analysis showed a significantly higher patient satisfaction and a significant reduction of pain after TAI treatment (p-value < 0.001 for both comparison). Specifically, as shown in Fig. 1, VAS for satisfaction mean scores increased of 3.6 points (SD 4.4) at follow-up, starting from a mean of 3.7 points at baseline (Table 2). As shown in Fig. 2, VAS for pain mean scores decreased of 1.8 points (SD 2.3) at follow-up, starting from a mean of 7.5 points at baseline (Table 2). Similarly, as depicted in Fig. 3, the LARS score decreased significantly from a mean baseline score of 21.9 to a mean follow-up score of 12.7 (p-value < 0.001, Table 2). Overall, after 12 months of using the TAI system, 75% of patients reported that they were more satisfied, 56.3% reported that pain decreased, and 78.1% reported that LARS-like symptoms decreased (Table 3). An additional analysis was performed to evaluate the impact of prior surgery on these outcomes, but no statistical significance was observed (satisfaction, *p* = 0.542; pain, *p* = 0.682; LARS, *p* = 0.660).

**Table 2:** Summary statistics for VAS and LARS scores by visit

		Visit				
		Baseline *N* = 32	Follow-up (FU) *N* = 32		Difference FU - Baseline	*p*-value^*^
VAS for Satisfaction	Mean (IQR)	3.7 (1.5,6.0)	7.3 (6.5,9.0)		3.6 (1.0,7.0)	
	Median	3.0	7.0		4.0	**< 0.001**
	Min, Max	0,10	0,10		-7,10	
VAS for Pain	Mean (IQR)	7.5 (7.0,10)	5.7 (4.5,7.0)		-1.8 (-3.0, 0)	
	Median	9.0	6.0		-2.0	**< 0.001**
	Min, Max	0,10	0,10		-7,3	
LARS score	Mean (IQR)	21.9 (12.0,30.0)	12.7 (4.5,19.0)		-9.3 (-20.5, -1.5)	
	Median	23.0	12.0		-5.0	**< 0.001**
	Min, Max	5,46	0,36		-37,16.0	

^*^ Signed rank sum test

**Table 3:** Frequency distribution of VAS and LARS categorized score changes at follow-up

	Score change		
	< 0	0	> 0
VAS for Satisfaction	6 (18.8)	2 (6.3)	24 (75.0)
VAS for Pain	18 (56.3)	11 (34.4)	3 (9.4)
LARS	25 (78.1)	2 (6.3)	5 (15.6)

 An improvement in patients’ QoL was also observed following TAI treatment. Indeed, most of the responses to the EHP-5 questionnaire significantly changed between the two time points [p = 0.002 for EHP-5(1), p = 0.03 for EHP-5(4) and p < 0.001 for EHP-5(5)]. As reported in Table 4, 40% of patients changed the response to the question regarding walking difficulties because of pain [EHP-5 (1)] from “often/always” at baseline to “never/rarely” at follow-up and 35% to “sometimes”. Some patients also reported that they felt that others did not understand what they were going through [EHP-5 (4)]; at baseline, 14 of them reported “never/very rarely”,” while 11 and 7 reported “sometimes” and “often/always”,” respectively. At follow-up, 72.7% of patients changed from “sometimes” to “never/very rarely” and 1 patient (14.3%) changed from “often/always” to “sometimes”. In response to the question “Do you feel that your appearance has been affected?” [(EHP-5 (5)], at baseline, 19 patients answered “often/always”,” 12 “sometimes” and only 1 “never/rarely”; at follow-up, 83.3% of patients changed from “sometimes” to “never/very rarely” and 31.6% changed from “often/always” to “never/very rarely”. The items EHP-5(2) and EHP-5(3) did not significantly differ between the two timepoints.

**Table 4:** EHP-5 questionnaire between visit answers agreement. Questions are: EHP-5 (1): found it difficult to walk because of the pain?; (2): felt as though symptoms are ruling your life?; (3): had mood swings?; (4): felt others do not understand what you are going through?; (5): felt your appearance has been affected?

		Baseline	Follow-up, *N* (Row%)				
		Row Total	Never/Rarely	Sometimes	Often/Always	Kappa (95%CI)	*p*-value^*^
EHP-5 (1)‡	**Never/Rarely**	4	4 (100)	0	0		
	**Sometimes**	4	2 (50.0)	1 (25.0)	1 (25.0)		
	**Often/Always**	20	8 (40.0)	7 (35.0)	5 (25.0)	0.21 (0.01,0.40)	**0.002**
EHP-5 (2)	**Never/Rarely**	0	0	0	0		
	**Sometimes**	7	6 (85.7)	1 (14.3)	0		
	**Often/Always**	25	10 (40.0)	7 (28.0)	8 (32.0)	NA^***†***^	NA^***†***^
EHP-5 (3)	**Never/Rarely**	6	5 (83.3)	1 (16.7)	0		
	**Sometimes**	11	3 (27.3)	4 (36.4)	4 (36.4)		
	**Often/Always**	15	4 (26.7)	7 (28.0)	8 (32.0)	0.37 (0.11,0.62)	0.17
EHP-5 (4)	**Never/Rarely**	14	14 (100)	0	0		
	**Sometimes**	11	8 (72.7)	3 (27.3)	0		
	**Often/Always**	7	0	1 (14.3)	6 (85.7)	0.61 (0.40,0.83)	**0.03**
EHP-5 (5)	**Never/Rarely**	1	1 (100)	0	0		
	**Sometimes**	12	10 (83.3)	1 (8.3)	1 (8.3)		
	**Often/Always**	19	6 (31.6)	4 (21.1)	9 (47.4)	0.17 (0.03,0.28)	**< 0.001**

^*^ Symmetry test; ^***†***^ *NA* = Not Applicable; ‡ Four patients refrained from responding to this particular query due to restricted ambulation resulting from concurrent neurological conditions

The weighted kappa statistic indicated that the changes were due to the clinical benefits of treatment, with a correlation observed between changes in QoL and the use of TAI. Although the correlation was small, it was found to be significant and ranged from a minimum of k = 0.17 - assessed as a response to EHP-5 (5) - to k = 0.37 - assessed as a response to EHP-5 (3) - with the exception of the response to EHP-5 (4) at k = 0.61 (Table 4).

Patients who discontinued TAI or experienced minimal benefits were compared to successful responders. Factors such as age, type of bowel dysfunction, and concomitant treatments were analyzed, but no definitive predictors of failure were identified.

In terms of safety, no AEs were recorded at follow-up and only 2 patients (3.3%) prematurely discontinued the treatment due to the occurrence of symptoms (pain and hemorrhoids).

## Discussion

Endometriosis is a complex condition often associated with pelvic pain and infertility, impacting patients’ quality of life significantly [1–5, 9]. Surgical interventions, such as those for deep endometriosis, may lead to symptoms resembling Low Anterior Resection Syndrome (LARS), compounding patients’ challenges. LARS-like symptoms, i.e. defecation dysfunctions, in patients with endometriosis are often complex and characterized by slow transit constipation and/or obstructive disorders, urgency, incomplete defecation, tenesmus or continence impairment [10, 11, 14]. Pelvic floor dysfunction exacerbates these issues, contributing to pelvic pain syndromes and bowel dysfunction [8, 10, 11, 14, 15, 24–26]. Several therapies such as dietary changes, pharmacological interventions, and pelvic floor rehabilitation may be necessary for comprehensive management; among these transanal irrigation (TAI) has been shown to significantly improve bowel symptoms, quality of life and satisfaction in patients with LARS-like bowel dysfunction [17–19, 27]. In the context of endometriosis, TAI seems to offer promising possibilities for symptom relief and improved pelvic floor rehabilitation, laying the foundation for the present pilot investigation.

In the clinical cases examined in this study, TAI treatment was indeed able to significantly improve women’s self-image, as well as physical activities typically impaired by pain, such as walking. The pain relief observed in more than half of the patients should not be underestimated, as it can lead to an improvement in social, occupational, and personal status. Accordingly, 75% of patients reported to be highly satisfied after 12 months of TAI treatment, representing an extremely valuable achievement, especially in the context of endometriosis management.

This positive impact on daily life and self-perception is consistent with the literature reporting on the QoL of patients with LARS and functional bowel disorders treated with TAI. Indeed, there is a growing number of studies reporting significantly improved QoL scores – assessed with disorder-specific questionnaires (e.g., PAC-QoL, FI-QoL, SF-36) – following the use of TAI [17, 18, 28–31]. Interestingly, the literature evaluating the efficacy of TAI in the treatment of LARS patients [18, 28] reports LARS scores around 12.0 after treatment, which ultimately confirms the results observed in the present study. Indeed, at follow-up, it was reported a mean LARS score of 12.7, a value that falls within the “no LARS” range, where few or no symptoms occur.

The TAI system proved to be an easy-to-use device, as only 3.3% of patients discontinued treatment due to technical difficulties in using the device. It can even be used at home, if necessary, with the help of a family member or caregiver, without any safety issues. Proof of the safety of the device is that only 3.3% of patients discontinued treatment due to the onset of symptoms and no AEs were recorded at follow-up. The observed dropout rate of 26.7% is similar to the TAI dropout rates of 18% and 23% reported in the recent literature on the use of the same TAI system for the treatment of LARS symptoms [18, 28].

This study represents the first analysis of the use of TAI in women afflicted with both endometriosis and LARS-like symptoms. The results of this retrospective analysis suggest the safe and effective use of the TAI system as a strategy to alleviate symptoms and improve QoL in women with endometriosis and bowel disease. Study limitations are related to the retrospective nature of its design and to the relatively small number of patients completing the follow-up questionnaire; additionally, the use of the LARS score, primarily designed to assess bowel dysfunction severity rather than treatment outcomes, may have underestimated the full impact of TAI treatment. Nonetheless, the findings presented here serve as an initial step towards conducting larger-scale studies, aiming to validate and build upon the promising outcomes observed.

## Conclusions

To the best of our knowledge, the results of the present study are the first evidence of the effectiveness of TAI in improving pain-related symptoms and QoL in patients with endometriosis in combination with bowel dysfunction. TAI was shown to provide significant improvements in pain relief, LARS-like symptoms, quality of life and satisfaction in both operated and non-operated patients. When offered to patients with endometriosis and bowel dysfunction, TAI appears to be a valuable strategy for reducing pelvic floor stress. Further studies should be conducted to determine whether TAI can also be useful during and/or after pelvic floor rehabilitation as a maintenance therapy and for other specific pathologies with similar symptoms.

## Data Availability

The datasets generated during and/or analysed during the current study are available from the corresponding author on reasonable request.
